# Supplementary material for: Establishing the effects of mesoporous silica nanoparticle properties on in vivo disposition using imaging-based pharmacokinetics
Source: Nat Commun. 2018 Oct 31;9:4551. doi: 10.1038/s41467-018-06730-z (PMC6208419; doi:10.1038/s41467-018-06730-z)
Supplement: Supplementary file 1 — Supplementary Information [file 41467_2018_6730_MOESM1_ESM.pdf]

Supplementary Information

Establishing the effects of mesoporous silica nanoparticle properties on *in vivo* disposition using imaging-based pharmacokinetics

Prashant Dogra†, Natalie L. Adolphi†, Zhihui Wang†, Yu-Shen Lin, Kimberly S. Butler, Paul N. Durfee, Jonas G. Croissant, Achraf Noureddine, Eric N. Coker, Elaine L. Bearer, Vittorio Cristini*, C. Jeffrey Brinker*

†These authors contributed equally to this work.

*Email: vcristini@houstonmethodist.org; cjbrink@sandia.gov

Supplementary Figures

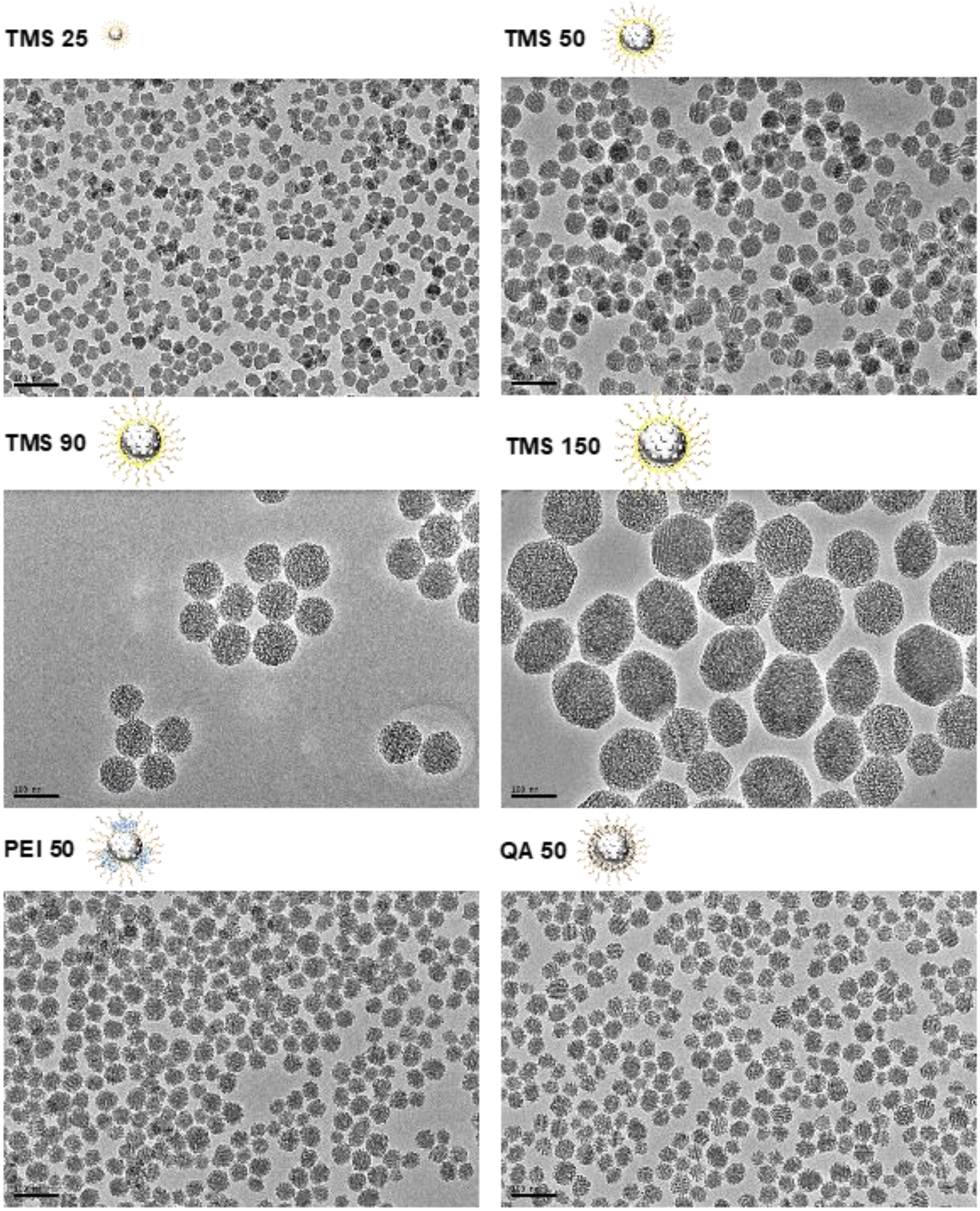

Supplementary Figure 1 | MSN core size characterization. Representative TEM images of the surface engineered MSNs used in the study. Scale bars: 100 nm.

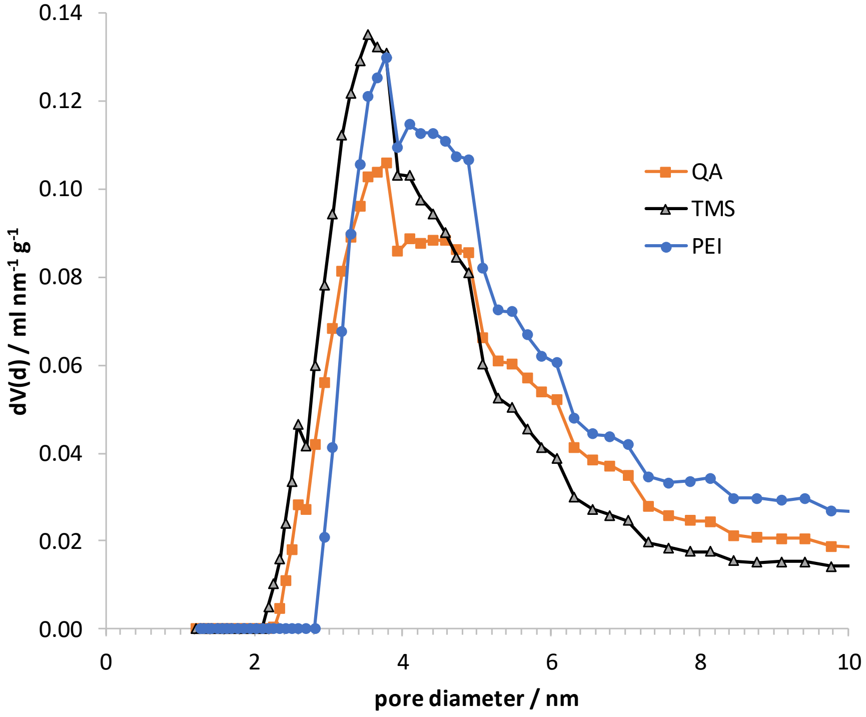

Supplementary Figure 2 | MSN pore size characterization. Pore diameter (nm) distributions calculated from the N_2 sorption data using Non-Local Density Functional Theory assuming a silica surface and cylindrical pores.

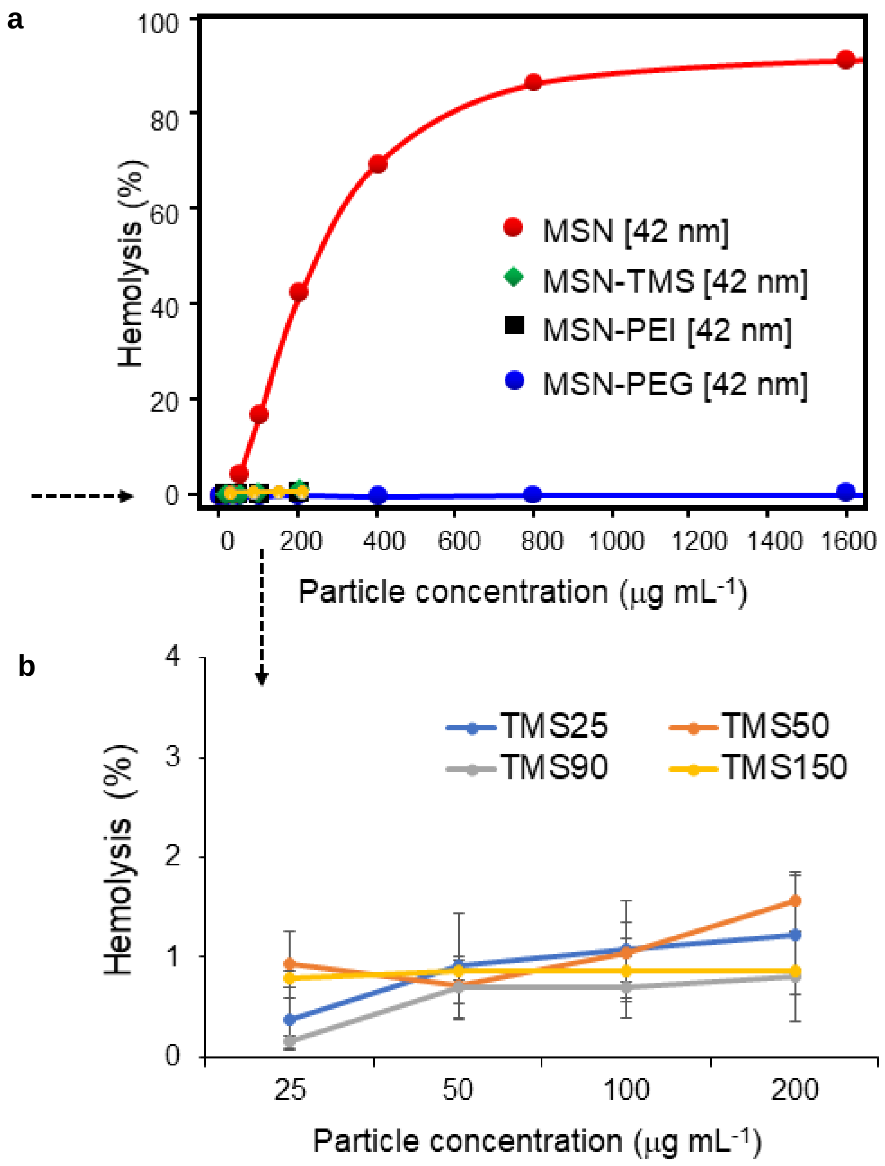

Supplementary Figure 3 | Hemolytic activity. Percentage hemolysis of RBCs incubated with **a**) 42-nm sized MSNs, with and without surface coatings (Lin et al.[1], Townson et al.[2]), and **b**) PEG-TMS coated MSNs of different sizes, at varying concentrations ranging from 3.125 to 1600 µg ml^{-1}. Data represents mean ± s.d. from atleast three independent experiments. Data in **b** is also shown in **a** for comparison (as indicated by dotted black arrows).

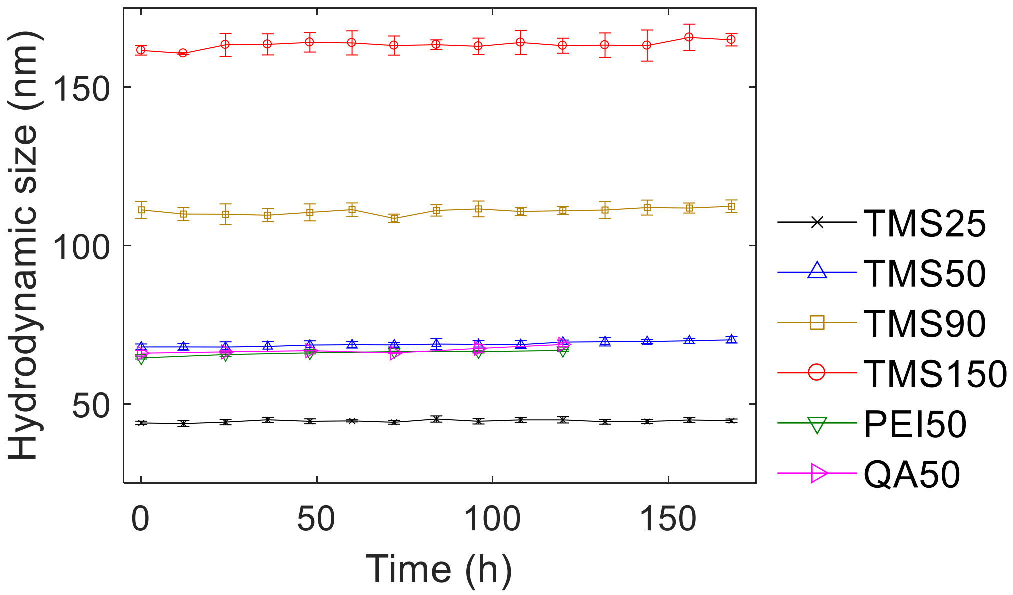

Supplementary Figure 4 | Hydrodynamic stability of MSNs. Time-dependent stability of MSNs in 1x PBS at 25°C over a period of five to seven days. Data represents mean ± s.d., n = 3.

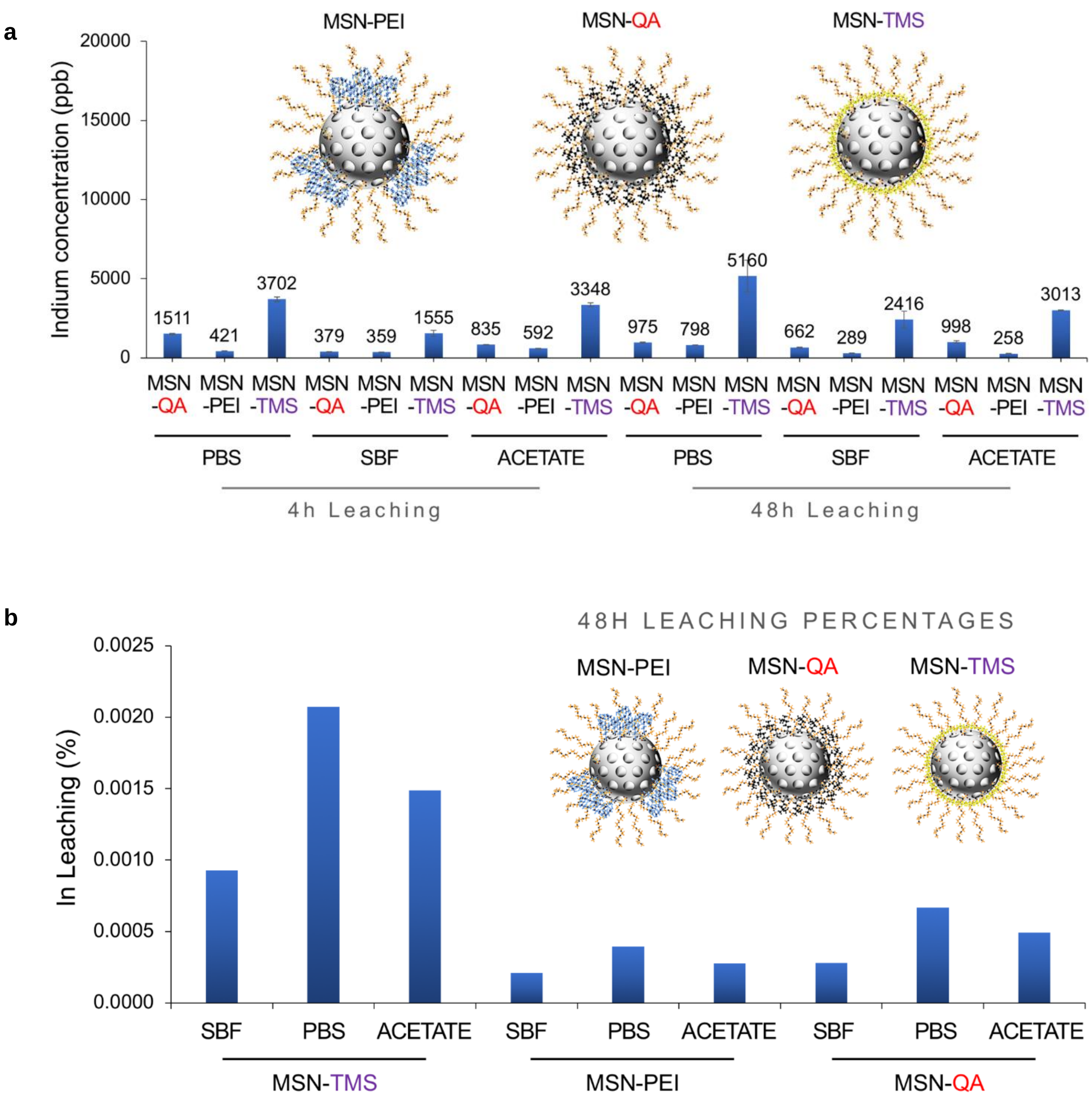

Supplementary Figure 5 | Indium (In) labeling stability testing. In leaching observed after incubation of 50 nm sized MSNs in physiologically relevant media (phosphate buffer saline (PBS), simulated body fluid (SBF), and acetate saline) at 37 °C using Graphite Furnace Atomic Absorption Spectrophotometry. Results are presented as **a,** In concentration in ppb of the media at 4 h and 48 h, and **b,** percentage of loaded In leached over 48 h. Error bars represent standard deviation over 3 measurements.

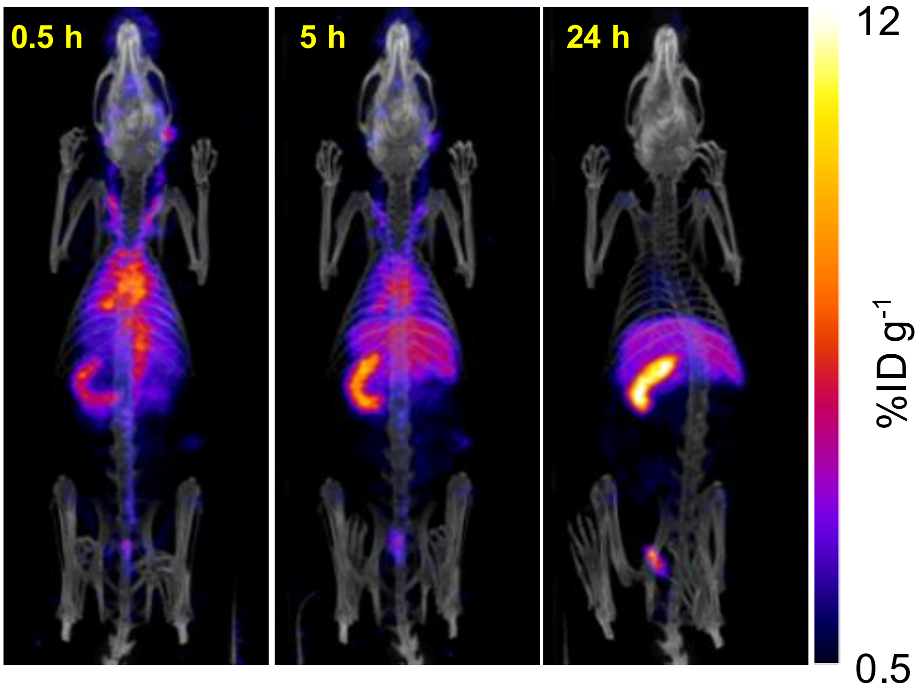

Supplementary Figure 6 | SPECT/CT imaging of TMS90 MSNs. Representative SPECT/CT images of a rat injected intravenously with TMS90 MSNs.

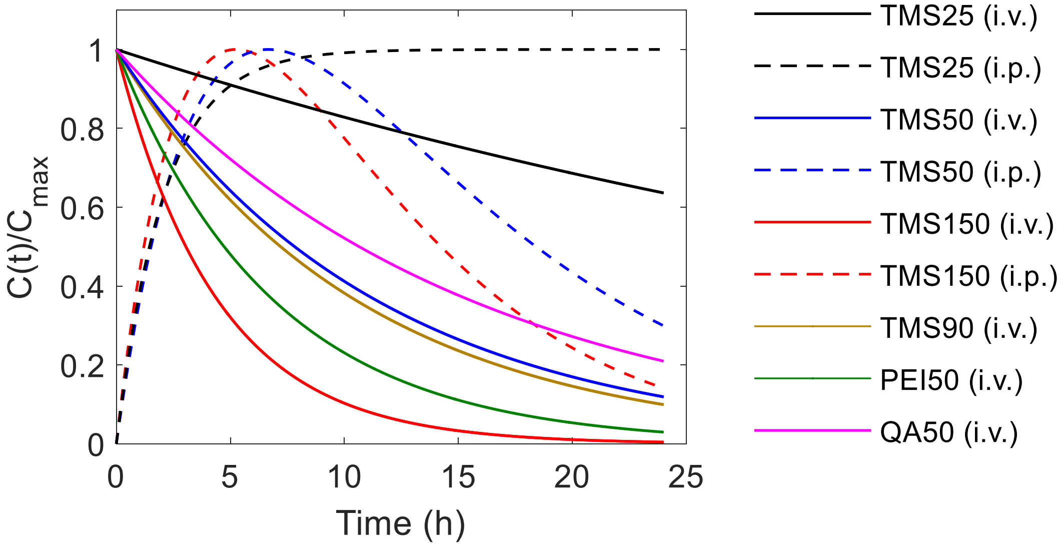

Supplementary Figure 7 | Kinetics of heart. Plot of predicted concentration ($C(t)$) normalized to predicted concentration maxima (C_{max}) of different MSNs in the heart.

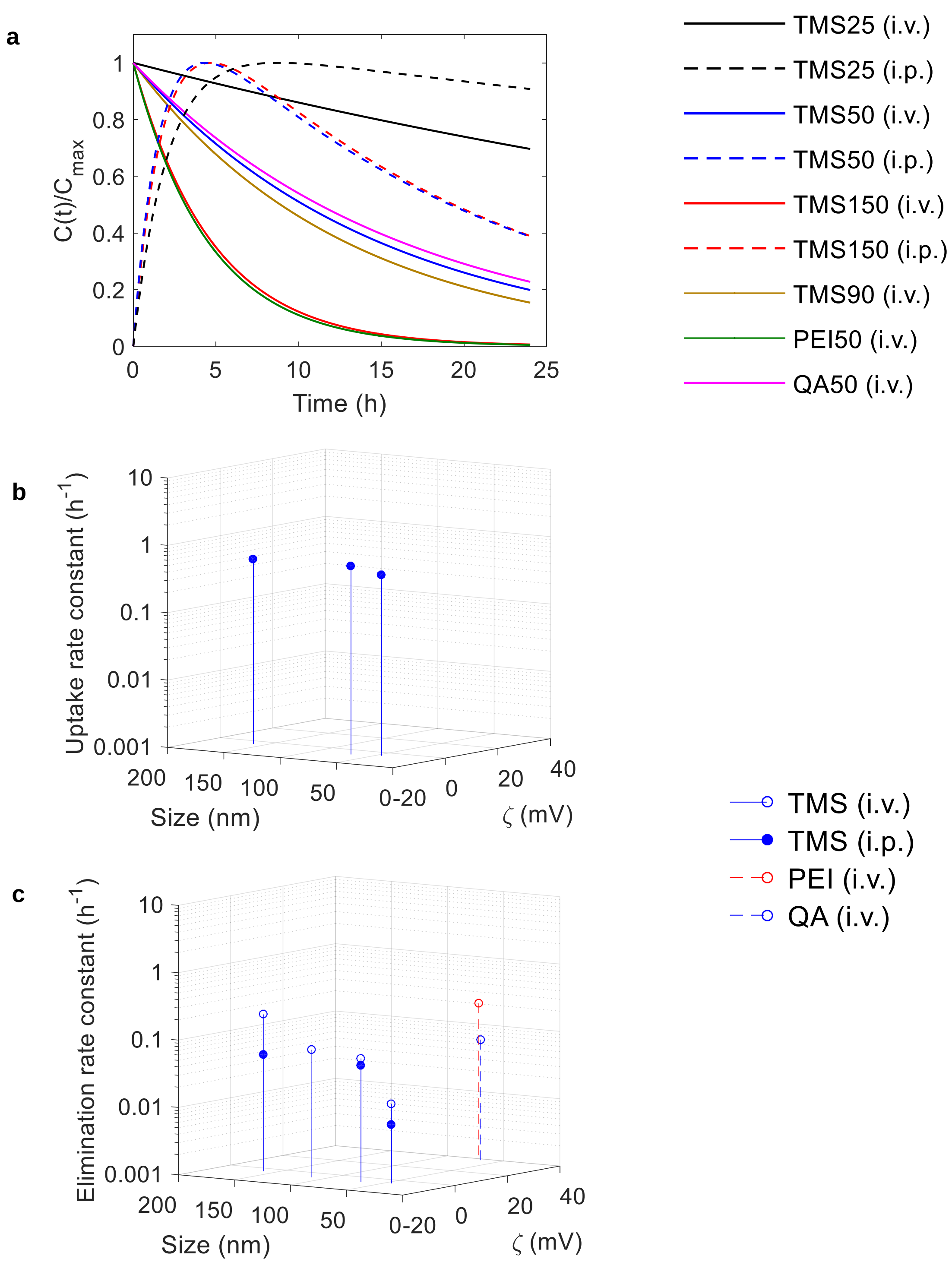

Supplementary Figure 8 | Kinetics of lungs. a, Plot of predicted concentration ($C(t)$) normalized to predicted concentration maxima (C_{max}) in the lungs. 3-D stem plots show **b**, uptake rate constants, k_{in} (h^{-1}), and **c**, elimination rate constants, k_{out} (h^{-1}), for the lungs in multiparameter space. Refer to Supplementary Table 4 and Supplementary Table 5 for more details.

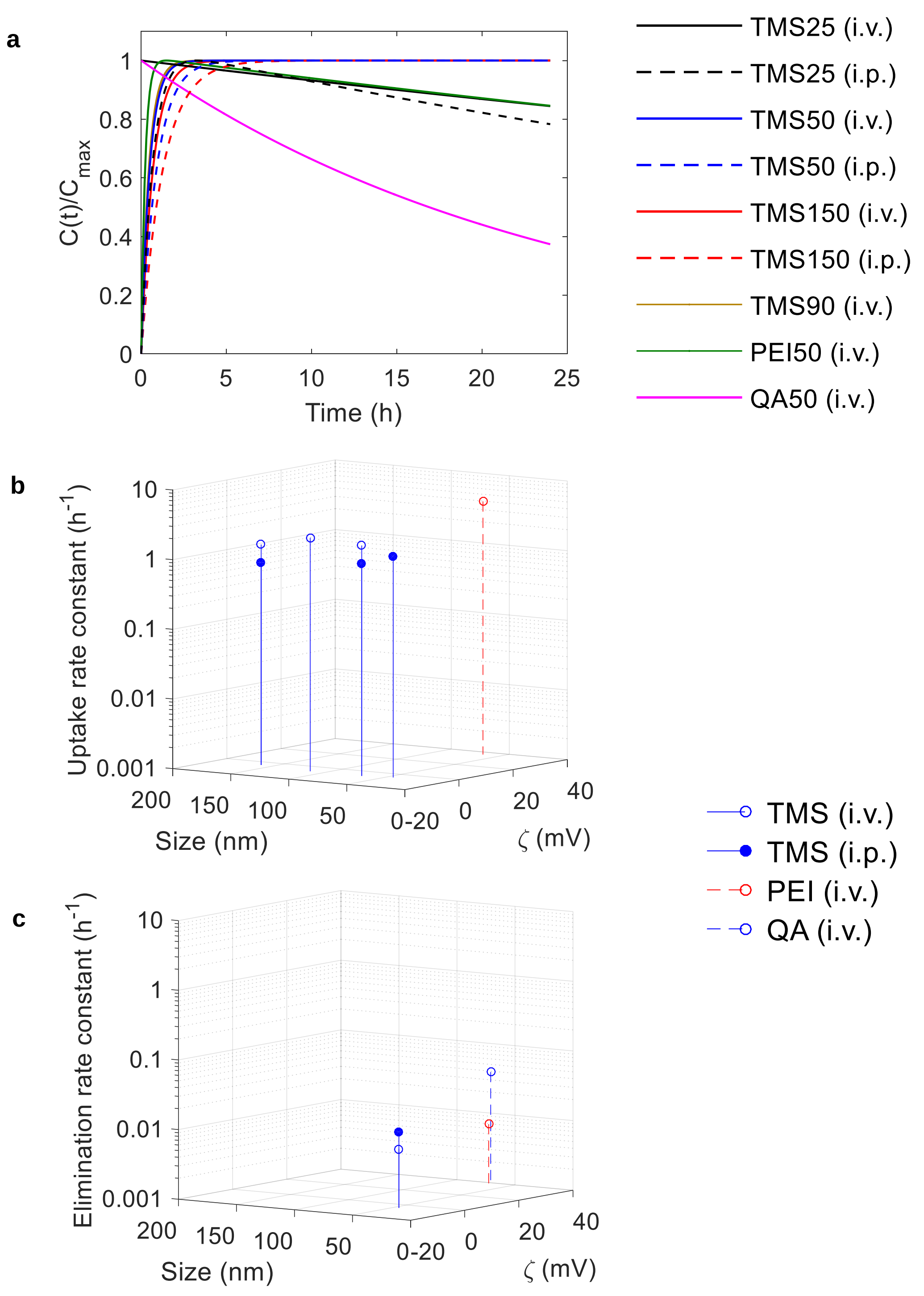

Supplementary Figure 9 | Kinetics of liver. a, Plot of predicted concentration ($C(t)$) normalized to predicted concentration maxima (C_{max}) in the liver. 3-D stem plots show **b**, uptake rate constants, k_{in} (h^{-1}), and **c**, elimination rate constants, k_{out} (h^{-1}), for the liver in multiparameter space. Refer to Supplementary Table 4 and Supplementary Table 5 for more details.

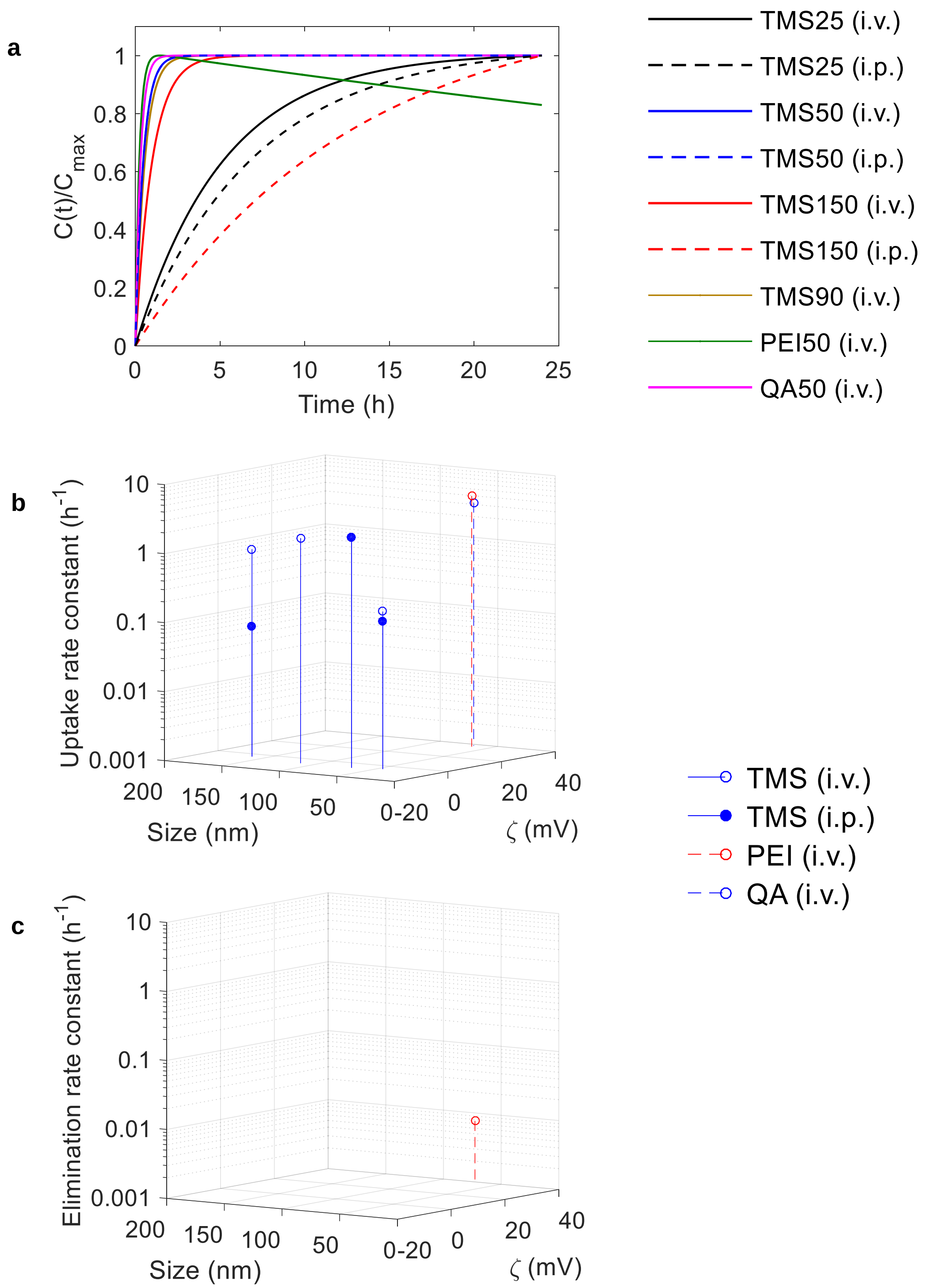

Supplementary Figure 10 | Kinetics of spleen. a, Plot of predicted concentration ($C(t)$) normalized to predicted concentration maxima (C_{max}) in the spleen. 3-D stem plots show **b**, uptake rate constants, k_{in} (h^{-1}), and **c**, elimination rate constants, k_{out} (h^{-1}), for the spleen in multiparameter space. Refer to Supplementary Table 4 and Supplementary Table 5 for more details.

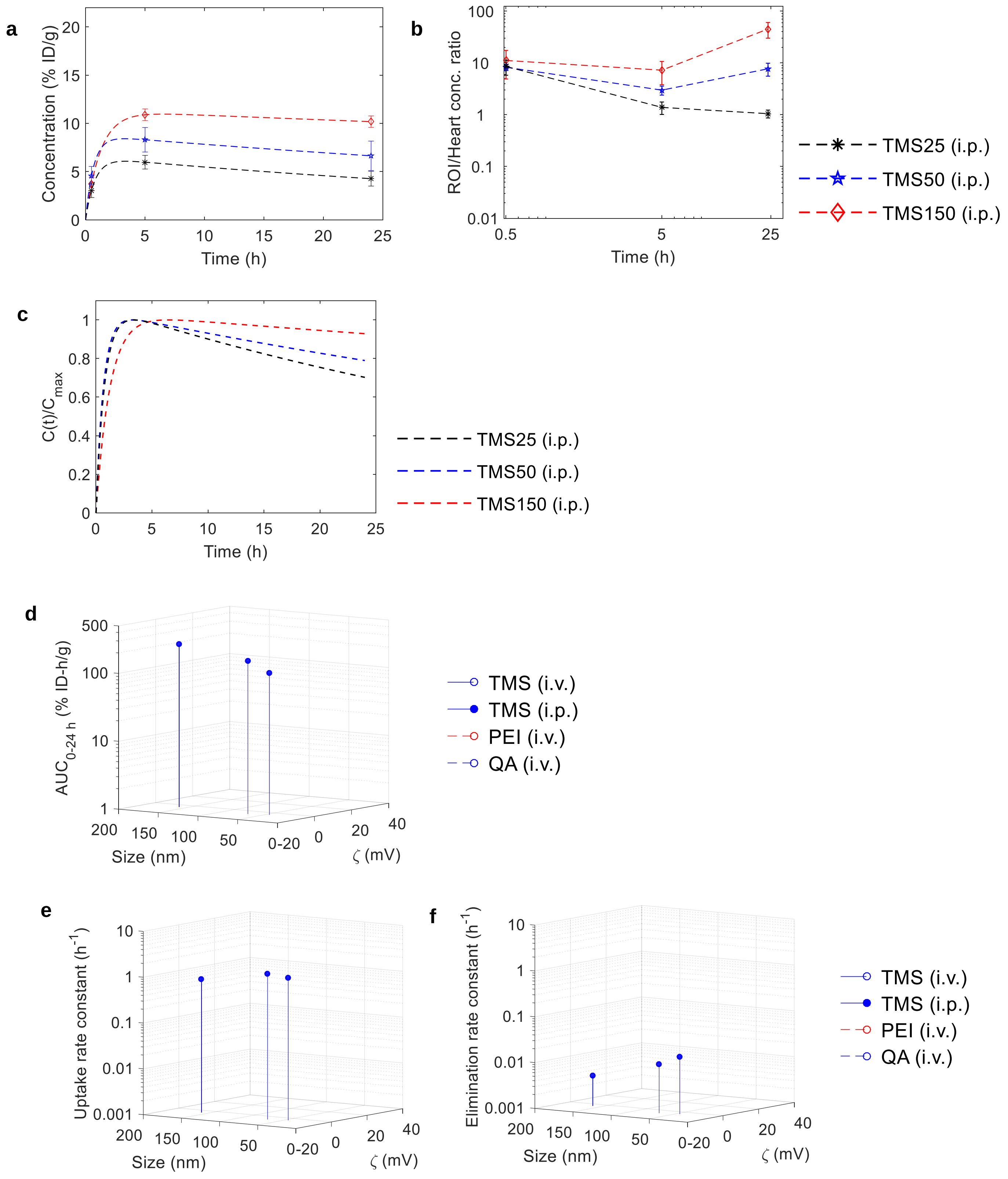

Supplementary Figure 11

Supplementary Figure 11 | Kinetics of thoracic lymph nodes. a, Plot of nonlinear regression of equation (4) to the concentration-time data of MSNs in thoracic lymph nodes. **b**, Observed concentration of thoracic lymph nodes normalized to concentration of heart (substitute for plasma) is shown over time on a log-log plot. **c**, Plot of predicted concentration ($C(t)$) normalized to predicted concentration maxima (C_{max}) in the thoracic lymph nodes. 3-D stem plots show **d**, area under the concentration-time curve ($AUC_{0-24\,h}$), **e**, uptake rate constants, k_{in} (h^{-1}), and **f**, elimination rate constants, k_{out} (h^{-1}), for the thoracic lymph nodes in multiparameter space. Refer to Supplementary Table 4 and Supplementary Table 5 for more details. Data represents mean ± s.d., *n* = 4 (except TMS50 (i.p.) and TMS25 (i.p.), where *n* = 3).

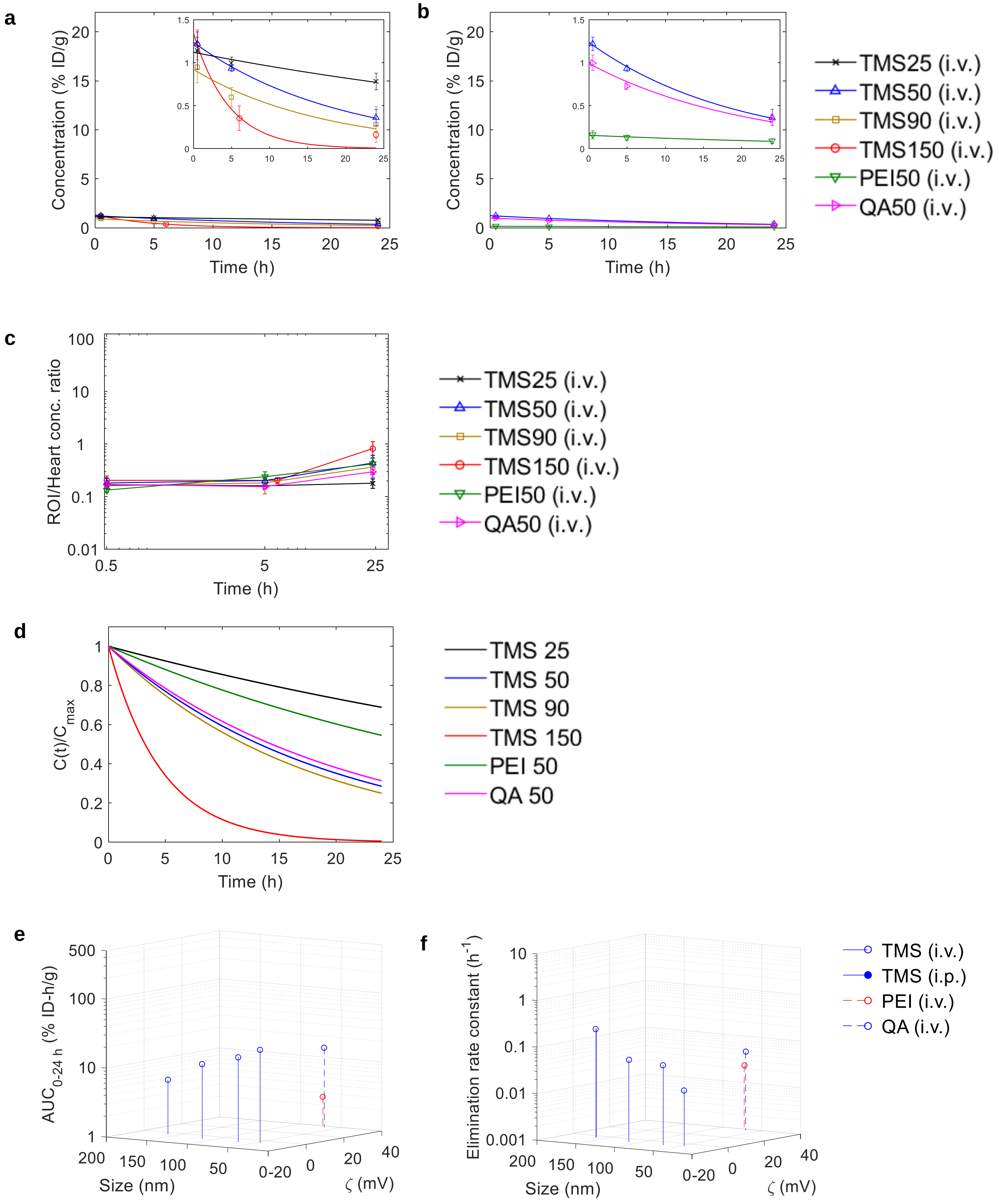

Supplementary Figure 12

Supplementary Figure 12 | Kinetics of abdominal aorta. a,**b**, Plots of nonlinear regression of equation (6) to the concentration-time data of MSNs in abdominal aorta. Fitted concentration-time curves demonstrate the effect of MSN size for TMS-modified MSNs (**a**) and surface chemistry and zeta potential for 50 nm diameter MSNs modified with TMS, QA, or PEI (**b**). The inset in (**a**,**b**) is a rescaled version of the figure for a clearer view. Note: Abdominal aorta was not analyzed as an ROI in the i.p. injection cases. **c**, Observed concentration of abdominal aorta normalized to concentration of heart (substitute for plasma) is shown over time on a log-log plot. **d**, Plot of predicted concentration $(C(t))$ normalized to predicted concentration maxima (C_{max}) in the abdominal aorta. 3-D stem plots show **e**, area under the concentration-time curve $(\mathrm{AUC}_{0-24\,\mathrm{h}})$ and **f**, elimination rate constants, $k_{out}\,(h^{-1})$, for the abdominal aorta in multiparameter space. Refer to Supplementary Table 4 and Supplementary Table 5 for more details. Data represents mean ± s.d., *n* = 4 (except TMS50 (i.p.) and TMS25 (i.p.), where *n* = 3).

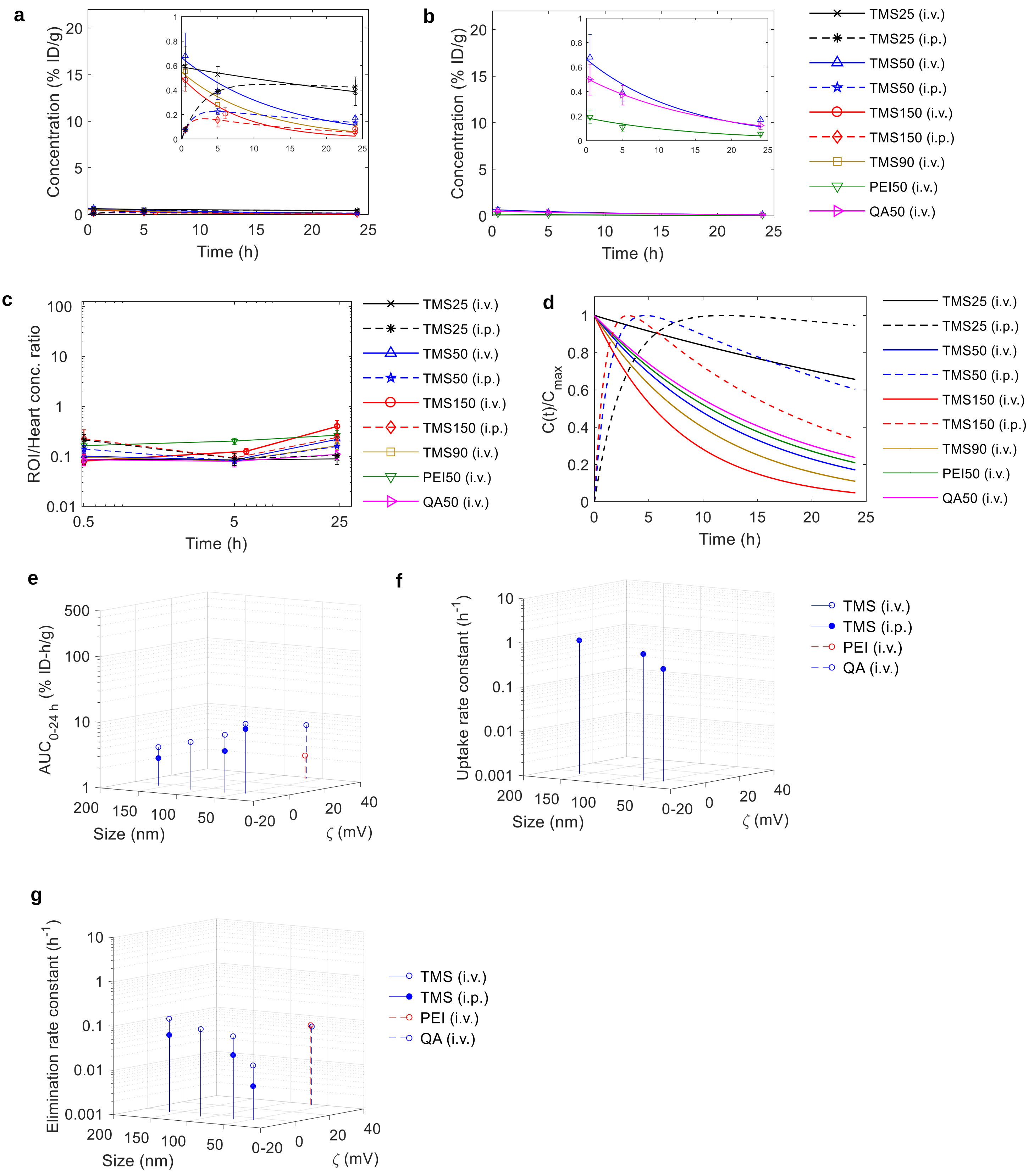

Supplementary Figure 13

Supplementary Figure 13 | Kinetics of muscles. a,**b**, Plots of nonlinear regression of equations (4,6) to the concentration-time data of MSNs in muscles. Fitted concentration-time curves demonstrate the effect of MSN size and route of administration for TMS-modified MSNs (**a**) and surface chemistry and zeta potential for 50 nm diameter MSNs modified with TMS, QA, or PEI (**b**). The inset in (**a**,**b**) is a rescaled version of the figure for a clearer view. Solid lines, i.v. cases (equation (6)); dotted lines, i.p. cases (equation (4)). **c**, Observed concentration of muscles normalized to concentration of heart (substitute for plasma) is shown over time on a log-log plot. **d**, Plot of predicted concentration $(C(t))$ normalized to predicted concentration maxima (C_{max}) in the muscles. 3-D stem plots show **e**, area under the concentration-time curve $(\mathrm{AUC}_{0-24\mathrm{h}})$ **f**, uptake rate constants, k_{in} (h^{-1}), and **g**, elimination rate constants, k_{out} (h^{-1}), for the muscles in multiparameter space. Refer to Supplementary Table 4 and Supplementary Table 5 for more details. Data represents mean ± s.d., *n* = 4 (except TMS50 (i.p.) and TMS25 (i.p.), where *n* = 3).

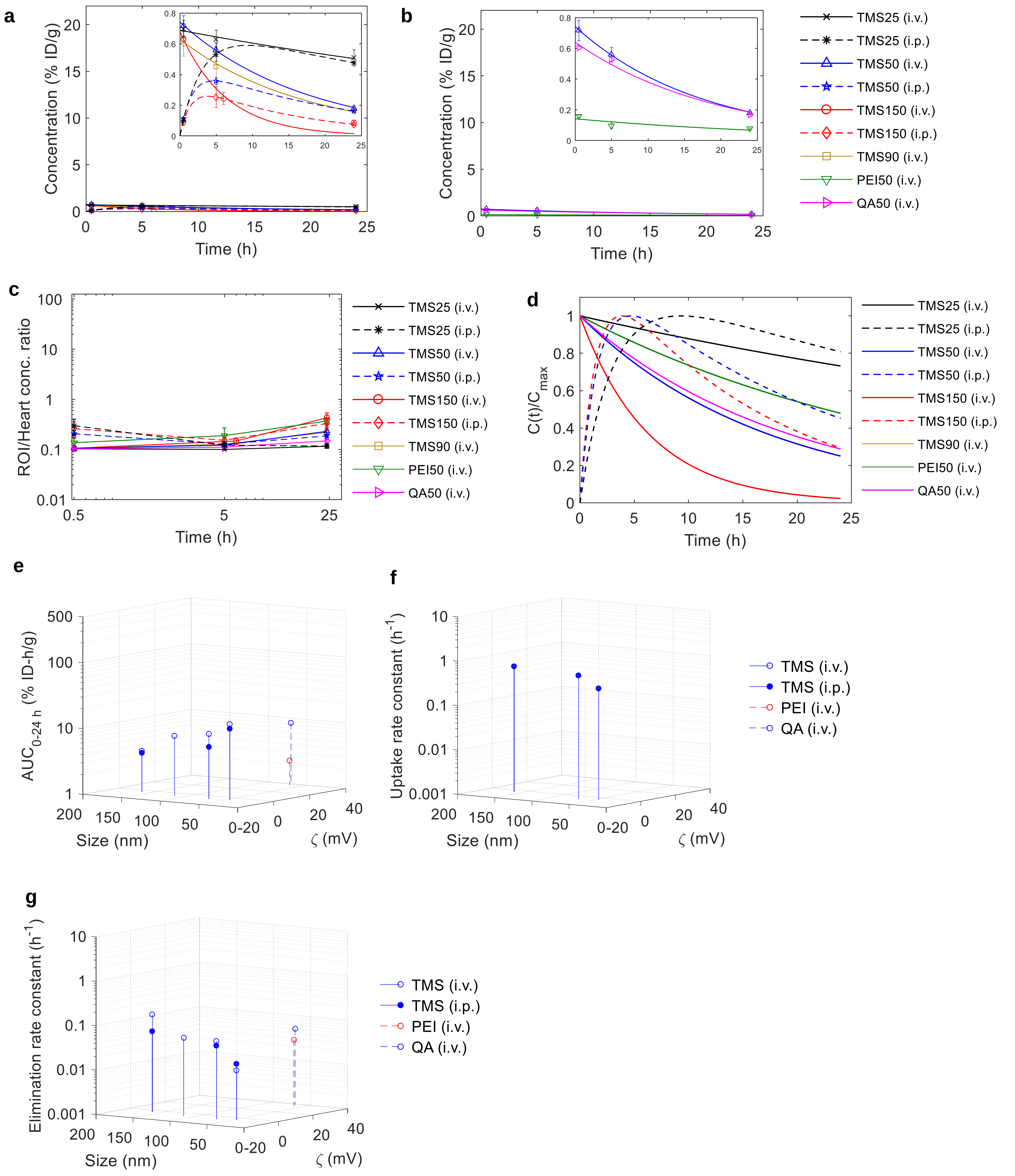

Supplementary Figure 14

Supplementary Figure 14 | Kinetics of brain. a,**b**, Plots of nonlinear regression of equations (4,6) to the concentration-time data of MSNs in brain. Fitted concentration-time curves demonstrate the effect of MSN size and route of administration for TMS-modified MSNs (**a**) and surface chemistry and zeta potential for 50 nm diameter MSNs modified with TMS, QA, or PEI (**b**). The inset in (**a**,**b**) is a rescaled version of the figure for a clearer view. Solid lines, i.v. cases (equation (6)); dotted lines, i.p. cases (equation (4)). **c**, Observed concentration of brain normalized to concentration of heart (substitute for plasma) is shown over time on a log-log plot. **d**, Plot of predicted concentration $(C(t))$ normalized to predicted concentration maxima (C_{max}) in the brain. 3-D stem plots show **e**, area under the concentration-time curve $(\mathrm{AUC}_{0-24\,\mathrm{h}})$ **f**, uptake rate constants, k_{in} (h^{-1}), and **g**, elimination rate constants, k_{out} (h^{-1}), for the brain in multiparameter space. Refer to Supplementary Table 4 and Supplementary Table 5 for more details. Data represents mean ± s.d., *n* = 4 (except TMS50 (i.p.) and TMS25 (i.p.), where *n* = 3).

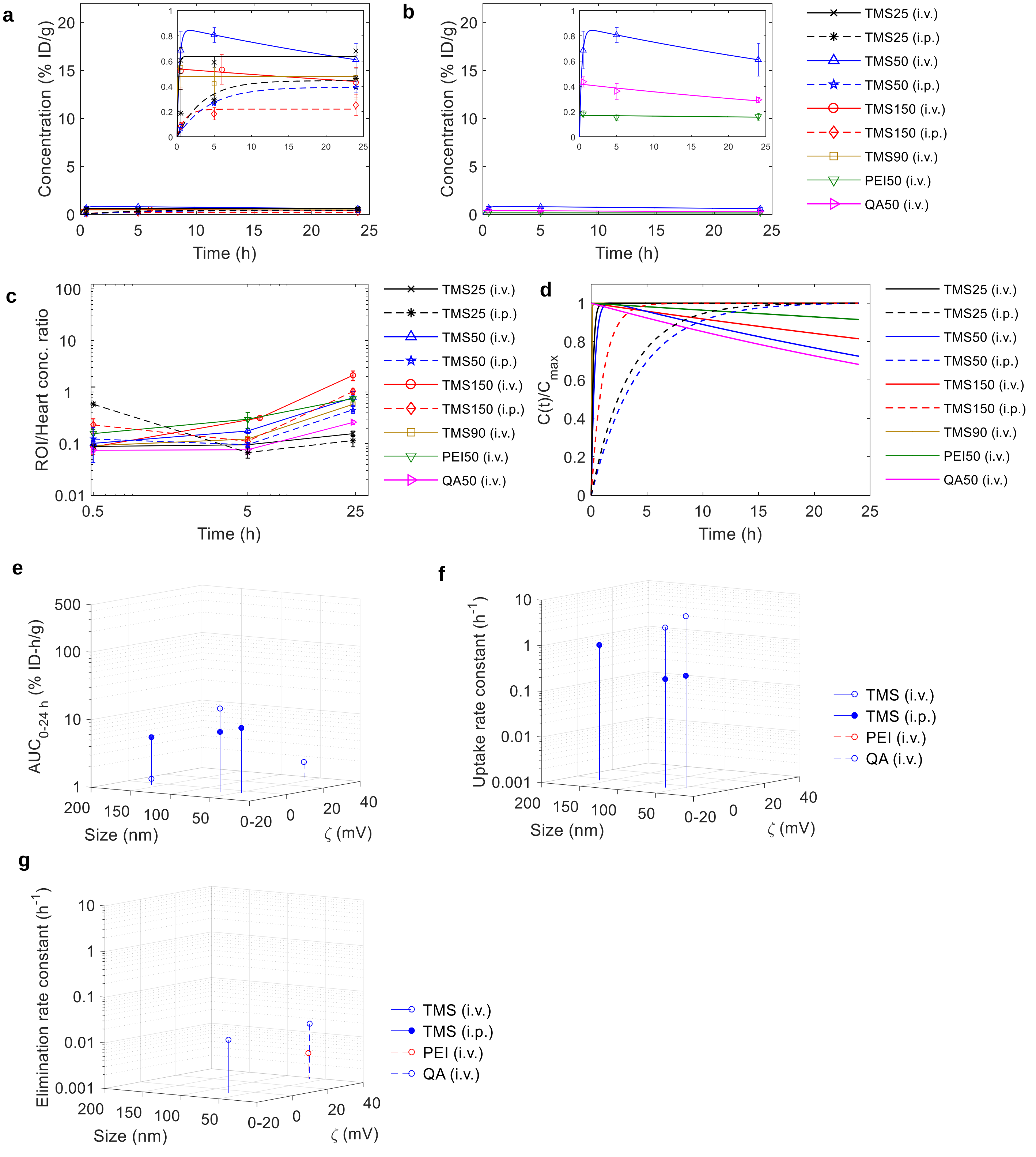

Supplementary Figure 15

Supplementary Figure 15 | Kinetics of joints. a,**b**, Plots of nonlinear regression of equations (4,5,6) to the concentration-time data of MSNs in joints. Fitted concentration-time curves demonstrate the effect of MSN size and route of administration for TMS-modified MSNs (**a**) and surface chemistry and zeta potential for 50 nm diameter MSNs modified with TMS, QA, or PEI (**b**). The inset in (**a**,**b**) is a rescaled version of the figure for a clearer view. Solid lines, i.v. cases (equation (6) for TMS150, PEI50, and QA50, equation (4) for TMS50, and equation (5) for TMS25 and TMS90); dotted lines, i.p. cases (equation (5)). **c**, Observed concentration of joints normalized to concentration of heart (substitute for plasma) is shown over time on a log-log plot. **d**, Plot of predicted concentration $(C(t))$ normalized to predicted concentration maxima (C_{max}) in the joints. 3-D stem plots show **e**, area under the concentration-time curve $(\mathrm{AUC}_{0-24\,\mathrm{h}})$ **f**, uptake rate constants, k_{in} (h^{-1}), and **g**, elimination rate constants, k_{out} (h^{-1}), for the joints in multiparameter space. Refer to Supplementary Table 4 and Supplementary Table 5 for more details. Data represents mean ± s.d., *n* = 4 (except TMS50 (i.p.) and TMS25 (i.p.), where *n* = 3).

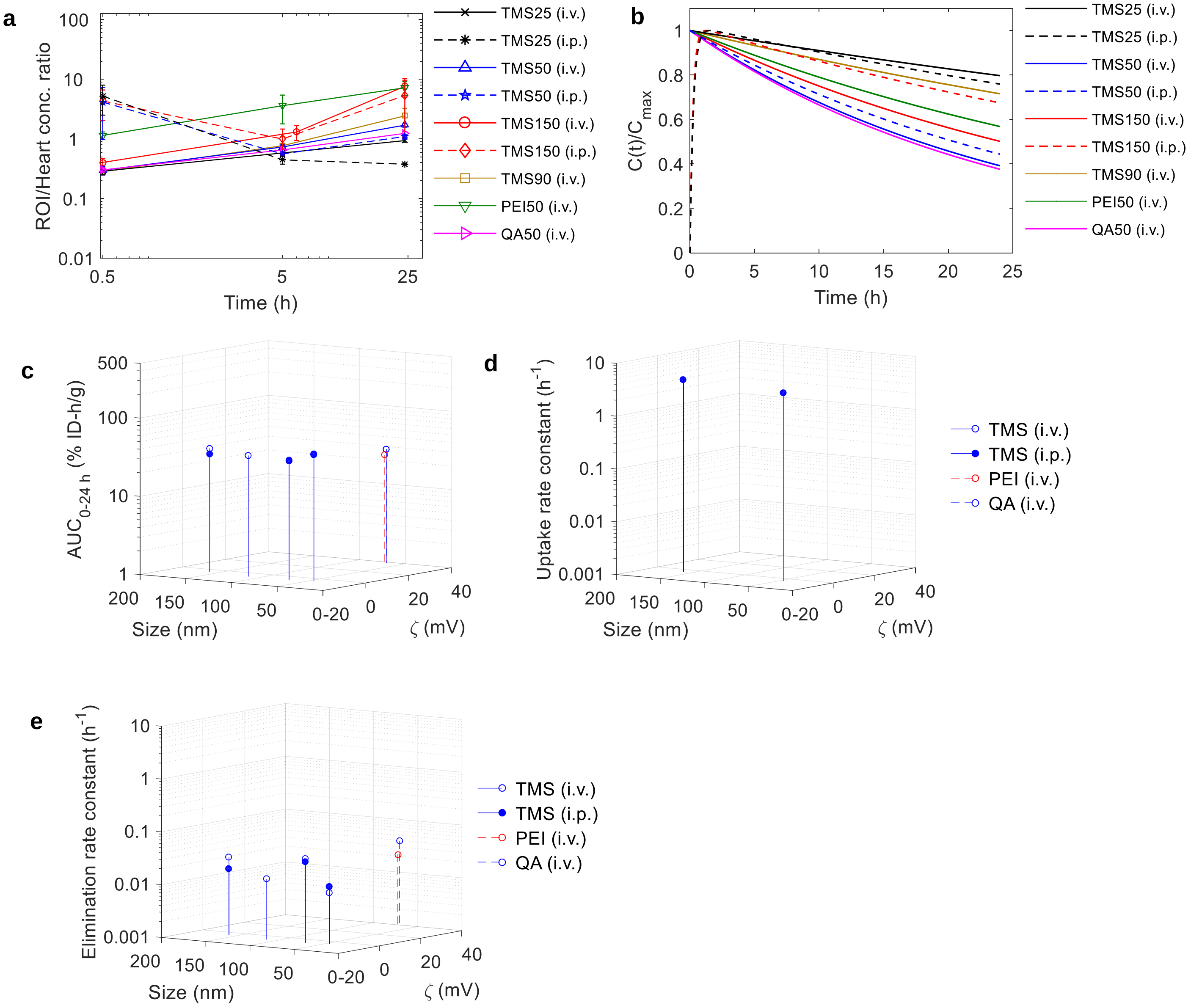

Supplementary Figure 16 | Kinetics of kidneys. a, Observed concentration of kidneys normalized to concentration of heart (substitute for plasma) is shown over time on a log-log plot. **b**, Plot of predicted concentration ($C(t)$) normalized to predicted concentration maxima (C_{max}) in the kidneys. 3-D stem plots show **c**, area under the concentration-time curve ($\mathrm{AUC}_{0-24\,\mathrm{h}}$), **d**, uptake rate constants, k_{in} (h^{-1}), and **e**, elimination rate constants, k_{out} (h^{-1}), for the kidneys in multiparameter space. Refer to Supplementary Table 4 and Supplementary Table 5 for more details. Data represents mean ± s.d., n = 4 (except TMS50 (i.p.) and TMS25 (i.p.), where n = 3).

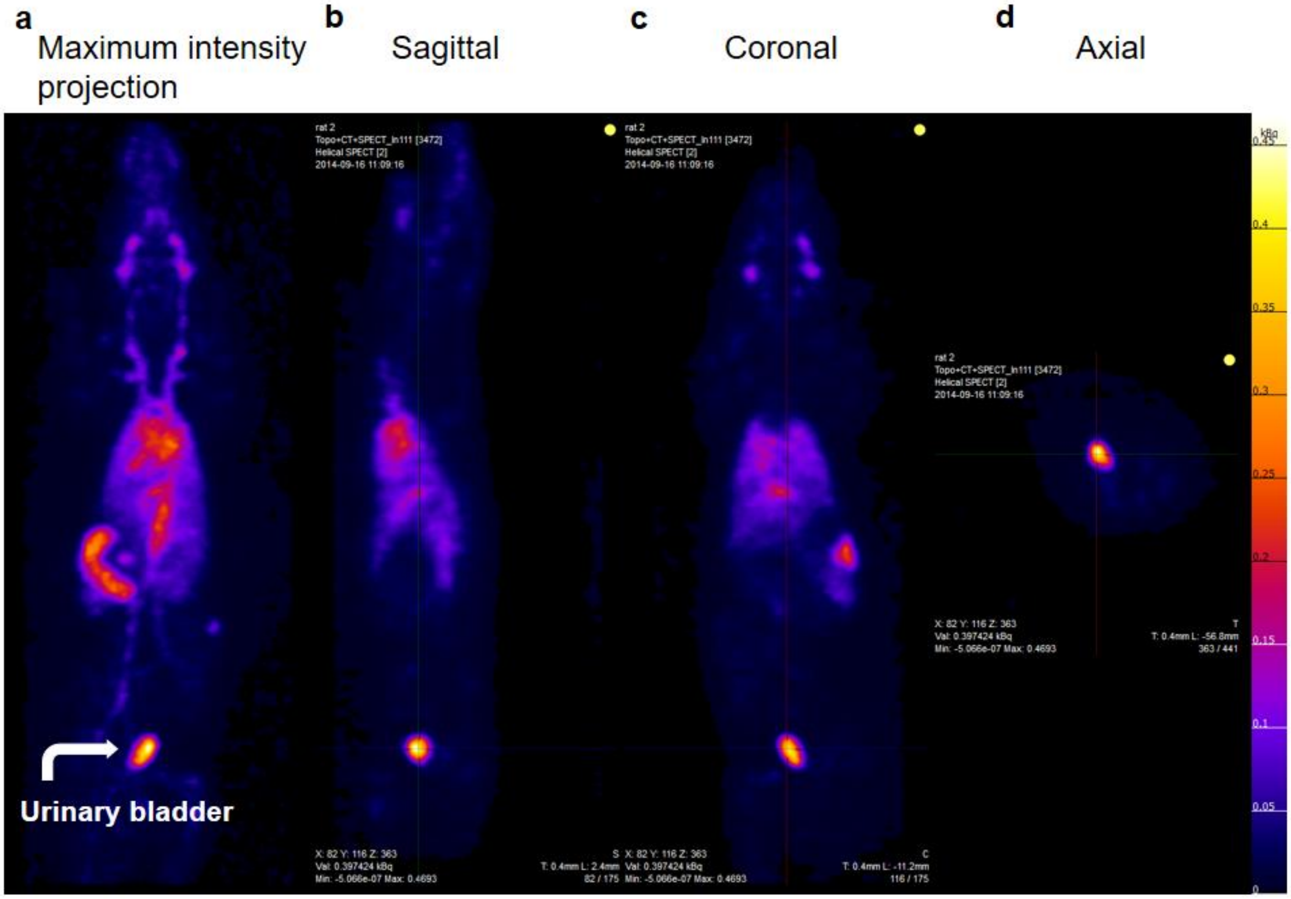

Supplementary Figure 17 | SPECT/CT images of MSN accumulation in urinary bladder. a, Representative maximum intensity projection image of a rat 30 min. post i.v. injection with TMS150 MSNs. **b**,**c**,**d**, Corresponding **b**, sagittal, **c**, coronal, and **d**, axial, cross-sectional images (0.4 mm slice thickness). The uniform distribution of radioactivity in the bladder from all three orientations indicates ^{111}In to be uniformly distributed throughout the bladder volume. Because the ^{111}In label was shown to be stable (Supplementary Fig. 5), this indicates that the signal in the bladder arises from MSNs or MSN fragments in urine and not from free ^{111}In label nor MSNs accumulated in the bladder wall via extravasation.

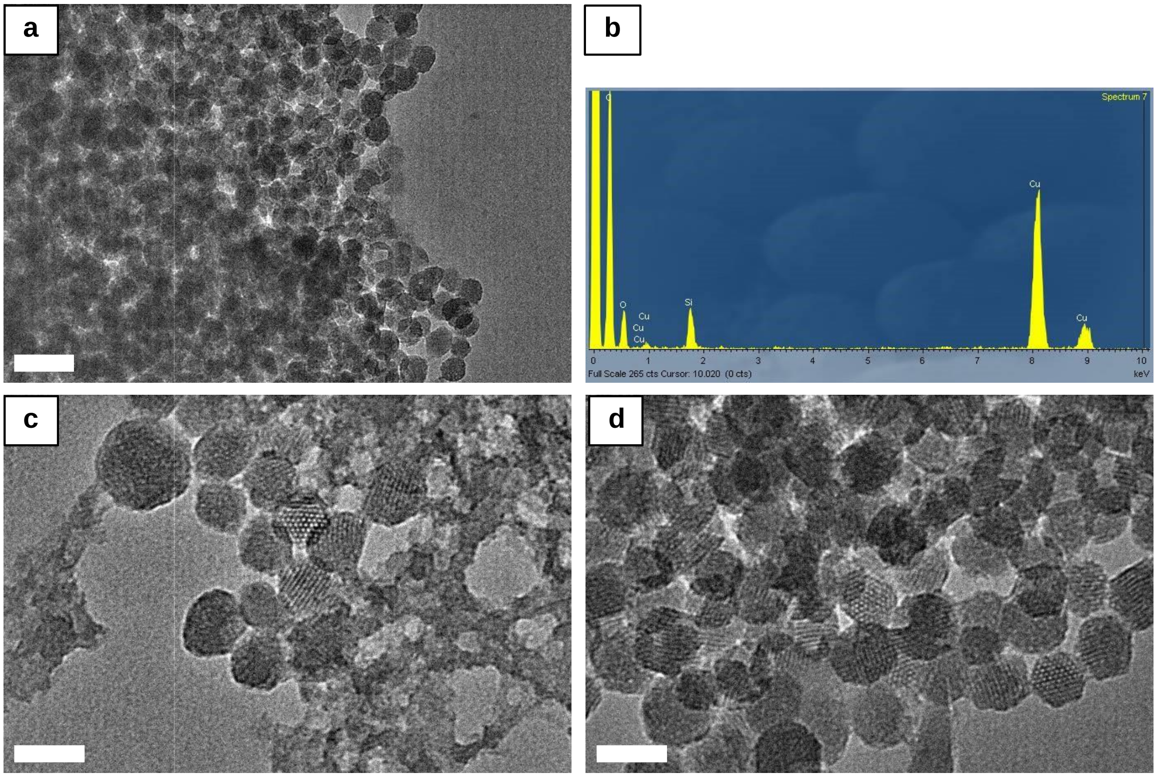

Supplementary Figure 18 | PEG-TMS MSNs in urine. Balb/C mice were injected with 1.5mg/mouse 50 nm PEG-TMS MSNs and urine was collected 24 hours post injection. **a**,**c**,**d,** TEM images of collected urine show the presence of particles retaining the features of the original injected MSNs. Scale bar is 100 nm in **a** and 50 nm in **c** and **d**. **b**, Spectral analysis confirmed the presence of silica in the particles present in urine.

a **Abdominal Aorta**

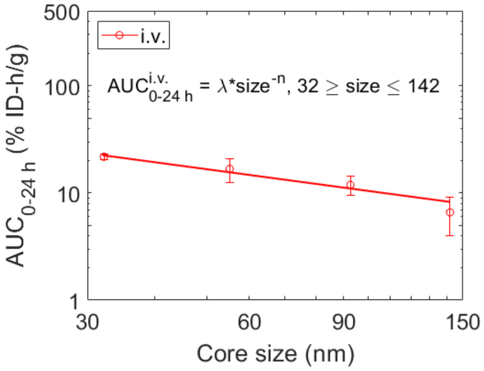

	Coefficient λ	Coefficient n
i.v.	232.6	0.67

b **Muscles**

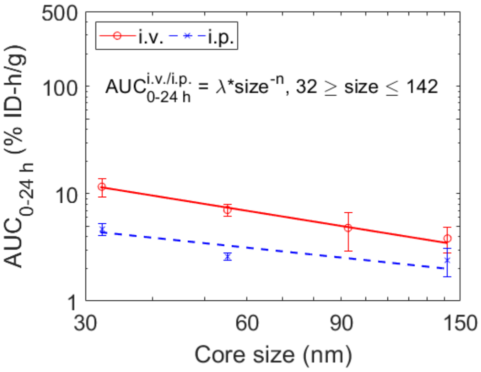

	Coefficient λ	Coefficient n
i.v.	188.2	0.81
i.p.	27.19	0.53

c **Brain**

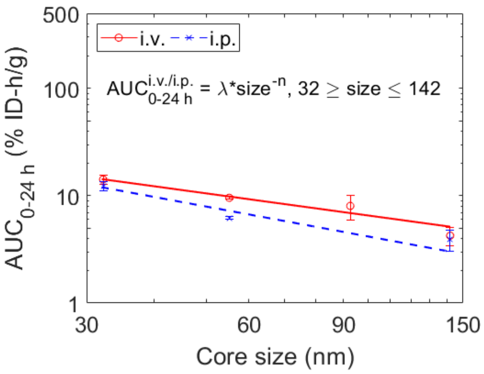

	Coefficient λ	Coefficient n
i.v.	153.8	0.69
i.p.	298.9	0.93

d **Thoracic lymph nodes**

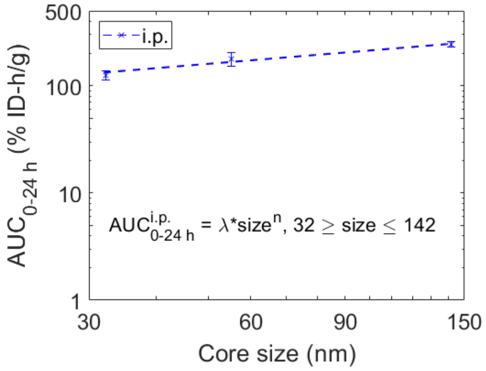

	Coefficient λ	Coefficient n
i.p.	31.68	0.41

e **Heart**

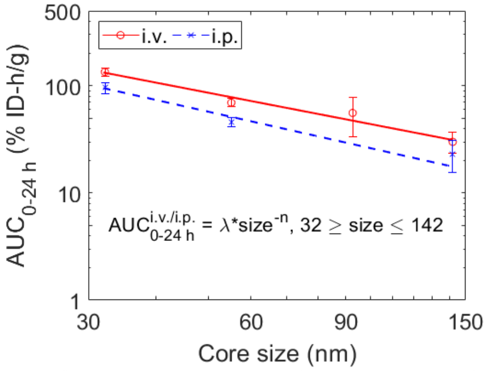

	Coefficient λ	Coefficient n
i.v.	3883	0.97
i.p.	4824	1.13

f **Lungs**

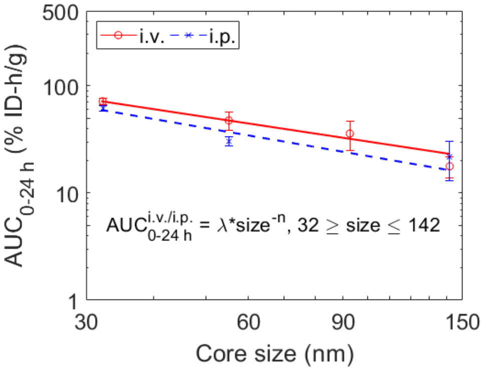

	Coefficient λ	Coefficient n
i.v.	1000	0.76
i.p.	1216	0.87

g **Liver**

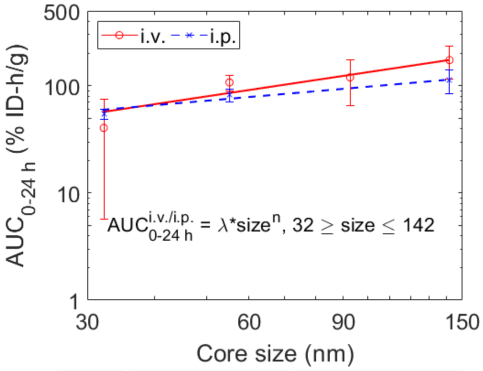

	Coefficient λ	Coefficient n
i.v.	4.2	0.75
i.p.	13.2	0.43

h **Spleen**

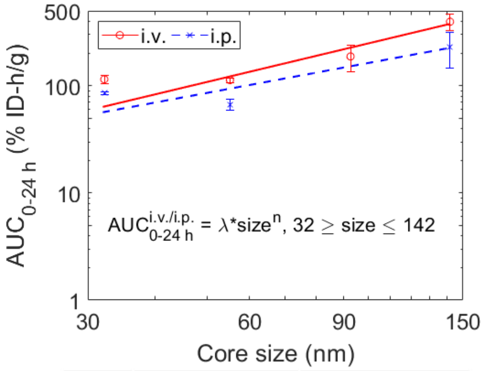

	Coefficient λ	Coefficient n
i.v.	1.002	1.19
i.p.	2.26	0.92

Supplementary Figure 19

Supplementary Figure 19 | Functional relationships between MSN core size and AUC. A function ($\mathrm{AUC}_{0-24\,\mathrm{h}} = \lambda \cdot \mathrm{size}^{n}$) was fit to obtain empirical relations for PEG-TMS coated MSNs in different ROIs. Data represents mean ± s.d., n = 4 (except TMS50 (i.p.) and TMS25 (i.p.), where n = 3). Results of fitted coefficient estimates are given in corresponding tables. For the studied size range (32 – 142 nm), the estimated values of power coefficient n exist in the range of 0.4 to 1.2 for sink-like organs, -0.5 to -0.9 for source-like organs, and ~ -1 for systemic circulation.

Supplementary Tables

Samples	NH_4OH concentration (M)	0.88 M ethanolic TEOS* (mL)	PEG-silane (μL)	TMS (μL)	PEI-silane (μL)	TMAC*-silane (μL)
**TMS25**	0.125	2.0	320	46.4	0	0
**TMS50**	0.250	2.5	400	58.0	0	0
**TMS90**	0.375	3.0	480	69.6	0	0
**TMS150**	0.500	3.0	480	69.6	0	0
**PEI50**	0.250	2.5	400	0	20	0
**QA50**	0.250	2.5	400	0	0	250

Supplementary Table 1 | Synthesis conditions of colloidally stable MSNs. *TEOS: tetraethyl orthosilicate, * TMAC: N-trimethoxysilylpropyl-N,N,N-trimethyl ammonium chloride

Sample	Average Pore Diameter (nm)	BET Surface area ($m^2\ g^{-1}$)	Density of In^{3+} (ions nm^{-2})
**QA**	3.8	533.9	5.8
**TMS**	3.5	569.5	6.9
**PEI**	3.8	589.6	4.0

Supplementary Table 2 | Textural properties of MSNs. Summary of the textural properties (pore size, surface area) of the three MSN types, and the calculated density of In^{3+} ions on the surface of each sample based on a correlation of elemental analysis and surface area. The surface density figures are similar to those established for gas adsorption on oxidic surfaces (e.g., between 6 and 7 N_2 molecules nm^{-2} at 77K). This points to a high density of chelating sites on the silica surface of these materials.

Medium	pH	Conductivity (mS/cm)
**Acetate saline**	5.2	15.3*
**Phosphate buffer saline (PBS)**	7.4	18.4
**Simulated body fluid (SBF)**	7.4	16.8

Supplementary Table 3 | Properties of physiologically relevant media. Properties of different buffers used in monitoring In leaching from MSNs. *Sodium chloride (NaCl) was added to the acetate saline buffer in order to have conductivity in the same range as other buffers (initially it was 8.2 mS/cm).

MSN ID	Heart			Lungs			Liver			Spleen			Thoracic lymph nodes		
	A	k_{in}	k_{out}	A	k_{in}	k_{out}	A	k_{in}	k_{out}	A	k_{in}	k_{out}	A	k_{in}	k_{out}
**TMS25 (i.v.)**	6.83 ± 0.37	-	0.01 ± 0.002	3.61 ± 0.13	-	0.01 ± 0.003	2.69 ± 0.16	-	1.51 ± 0.86	5.5 ± 0.4	0.58 ± 0.22	-	-	-	-
**TMS25 (i.p.)**	4.45 ± 0.26	0.45 ± 0.09	-	3.24 ± 0.21	0.45 ± 0.07	0.009 ± 0.004	2.75 ± 0.35	2.16 ± 0.78	0.01 ± 0.005	4.7 ± 0.22	0.16 ± 0.01	-	6.9 ± 1.5	1.5 ± 0.5	0.02 ± 0.006
**TMS50 (i.v.)**	7.14 ± 0.26	-	0.09 ± 0.01	4.05 ± 0.1	-	0.07 ± 0.01	4.58 ± 0.4	2.15 ± 0.36	-	4.8 ± 0.1	2.27 ± 0.36	-	-	-	-
**TMS50 (i.p.)**	26.1 ± 20.3	0.26 ± 0.05	0.09 ± 0.01	2.69 ± 0.36	0.61 ± 0.14	0.05 ± 0.004	3.58 ± 0.3	1.11 ± 0.17	-	4.32 ± 1.1	75.4 ± 75.2	-	8.9 ± 0.9	1.6 ± 0.3	0.01 ± 0.008
**TMS90 (i.v.)**	6.2 ± 0.4	-	0.11 ± 0.02	3.38 ± 0.26	-	0.08 ± 0.01	5.1 ± 1.15	12.1 ± 9.9	-	8.2 ± 1.1	1.7 ± 0.58	-	-	-	-
**TMS150 (i.v.)**	6.75 ± 0.5	-	0.23 ± 0.03	3.73 ± 0.2	-	0.21 ± 0.01	7.48 ± 1.32	1.82 ± 0.5	-	17.2 ± 1.5	1.01 ± 0.13	-	-	-	-
**TMS150 (i.p.)**	24.09 ± 12.9	0.27 ± 0.05	0.14 ± 0.01	4.67 ± 1.15	0.39 ± 0.16	0.08 ± 0.02	5.09 ± 0.74	0.69 ± 0.15	-	620 ± 601	0.07 ± 0.02	-	11.4 ± 0.2	0.79 ± 0.07	0.004 ± 0.0008
**PEI50 (i.v.)**	1.27 ± 0.16	-	0.15 ± 0.03	1.58 ± 0.32	-	0.17 ± 0.06	11.68 ± 0.46	4.4 ± 0.47	0.007 ± 0.002	13.1 ± 0.3	5.09 ± 1.1	0.009 ± 0.004	-	-	-
**QA50 (i.v.)**	6.18 ± 0.13	-	0.06 ± 0.0006	3.11 ± 0.13	-	0.06 ± 0.003	2.86 ± 0.12	-	0.04 ± 0.003	3.4 ± 0.1	3.3 ± 0.08	-	-	-	-

MSN ID	Brain			Abdominal aorta		Joints			Muscles			Kidneys			Total excretion	
	A	k_{in}	k_{out}	A	k_{out}	A	k_{in}	k_{out}	A	k_{in}	k_{out}	A	k_{in}	k_{out}	U_t	k_u
**TMS25 (i.v.)**	0.67 ± 0.02	-	0.01 ± 0.001	1.13 ± 0.02	0.02 ± 0.004	0.63 ± 0.02	-	16 ± 10.9	0.6 ± 0.03	-	0.02 ± 0.004	1.93 ± 0.07	-	0.009 ± 0.001	28.8 ± 23.7	0.58 ± 0.48
**TMS25 (i.p.)**	0.83 ± 0.2	0.35 ± 0.07	0.02 ± 0.009	-	-	0.33 ± 0.2	0.14 ± 0.09	-	4.9 ± 0.001	0.09 ± 0.002	0.07 ± 0.001	2.12 ± 0.1	14.8 ± 11.1	0.013 ± 0.002	100 ± 1e-6	0.001 ± 0.3e-3
**TMS50 (i.v.)**	0.74 ± 0.02	-	0.05 ± 0.004	1.24 ± 0.07	0.05 ± 0.01	0.87 ± 0.05	3.48 ± 0.71	0.015 ± 0.005	0.68 ± 0.11	-	0.08 ± 0.02	2.18 ± 0.13	-	0.039 ± 0.006	85.6 ± 14.3	0.025 ± 0.012
**TMS50 (i.p.)**	0.48 ± 0.04	0.6 ± 0.06	0.04 ± 0.004	-	-	0.39 ± 0.02	0.24 ± 0.02	-	0.18 ± 0.02	0.7 ± 0.1	0.04 ± 0.004	2.08 ± 0.53	-	0.03 ± 0.01	100 ± 5e-7	0.01 ± 0.5e-3
**TMS90 (i.v.)**	0.63 ± 0.06	-	0.06 ± 0.007	0.92 ± 0.08	0.05 ± 0.005	0.48 ± 0.06	-	42.7 ± 12.4	0.57 ± 0.03	-	0.13 ± 0.03	1.72 ± 0.24	-	0.014 ± 0.005	80.4 ± 19.5	0.79 ± 0.78
**TMS150 (i.v.)**	0.67 ± 0.02	-	0.16 ± 0.01	1.34 ± 0.11	0.22 ± 0.03	0.55 ± 0.02	-	0.01 ± 0.007	0.5 ± 0.06	-	0.13 ± 0.03	2.16 ± 0.08	-	0.029 ± 0.006	92 ± 7.6	0.016 ± 0.009
**TMS150 (i.p.)**	0.37 ± 0.05	0.68 ± 0.05	0.06 ± 0.006	-	-	0.23 ± 0.04	0.7 ± 0.23	-	0.22 ± 0.05	1.24 ± 0.3	0.06 ± 0.008	1.67 ± 0.17	6.3 ± 3	0.02 ± 0.01	100 ± 3e-7	0.014 ± 0.002
**PEI50 (i.v.)**	0.14 ± 0.005	-	0.03 ± 0.0006	0.15 ± 0.02	0.02 ± 0.004	0.17 ± 0.011	-	0.005 ± 0.002	0.19 ± 0.02	-	0.08 ± 0.03	1.36 ± 0.18	-	0.026 ± 0.01	25.4 ± 1.3	0.36 ± 0.26
**QA50 (i.v.)**	0.63 ± 0.007	-	0.05 ± 0.001	0.99 ± 0.03	0.04 ± 0.005	0.41 ± 0.02	-	0.01 ± 0.0009	0.53 ± 0.05	-	0.07 ± 0.02	1.87 ± 0.05	-	0.04 ± 0.002	77.4 ± 6.7	0.05 ± 0.007

Supplementary Table 4 | Individual animal non-linear regression analysis. Values of model parameter estimates, obtained by fitting the model equations to individual animal data, are presented as mean ± s.e.m. for various ROIs. Units of A, k_{in}, k_{out}, U_t, and k_u are %ID g^{-1}, h^{-1}, h^{-1}, %ID, and h^{-1}, respectively. (Note: Abdominal aorta and lymph nodes were not analyzed as an ROI in the i.p. and i.v. injection cases, respectively).

MSN ID	Heart				Lungs				Liver				Spleen				Thoracic lymph nodes			
	A	k_{in}	k_{out}	$RMSE$	A	k_{in}	k_{out}	$RMSE$	A	k_{in}	k_{out}	$RMSE$	A	k_{in}	k_{out}	$RMSE$	A	k_{in}	k_{out}	$RMSE$
**TMS25 (i.v.)**	6.86	-	0.018	0.008	3.6	-	0.01	0.009	2.67	-	0.007	0.14	6.0	0.19	-	1.4	-	-	-	-
**TMS25 (i.p.)**	4.40	0.48	-	0.79	3.07	0.48	0.007	-	2.64	1.48	0.01	-	4.82	0.14	-	0.9	6.54	1.29	0.01	-
**TMS50 (i.v.)**	7.09	-	0.088	0.06	4.02	-	0.06	0.32	4.58	2.01	-	0.56	4.81	2.17	-	1.1	-	-	-	-
**TMS50 (i.p.)**	12.005	0.2	0.1	-	2.51	0.6	0.05	-	3.57	1.1	-	0.36	2.80	2.16	-	1.2	8.81	1.49	0.01	-
**TMS90 (i.v.)**	6.09	-	0.09	0.28	3.35	-	0.07	0.01	5.08	2.23	-	0.14	7.94	1.82	-	1.3	-	-	-	-
**TMS150 (i.v.)**	6.72	-	0.22	0.17	3.73	-	0.21	0.23	7.47	1.44	-	0.3	17.22	1.01	-	1.1	-	-	-	-
**TMS150 (i.p.)**	10.66	0.23	0.15	-	1.85	0.54	0.05	-	4.91	0.78	-	0.37	17.57	0.07	-	1.8	11.35	0.8	0.004	-
**PEI50 (i.v.)**	1.26	-	0.14	0.18	1.66	-	0.22	0.5	11.67	4.3	0.007	-	13.06	4.3	0.008	-	-	-	-	-
**QA50 (i.v.)**	6.18	-	0.06	0.33	3.11	-	0.06	0.09	2.86	-	0.04	0.15	3.46	3.36	-	0.2	-	-	-	-

MSN ID	Brain				Abdominal aorta				Joints				Muscles				Kidneys				Total excretion		
	A	k_{in}	k_{out}	$RMSE$	A	k_{in}	k_{out}	$RMSE$	A	k_{in}	k_{out}	$RMSE$	A	k_{in}	k_{out}	$RMSE$	A	k_{in}	k_{out}	$RMSE$	U_t	k_u	$RMSE$
**TMS25 (i.v.)**	0.68	-	0.01	0.02	1.12	-	0.01	0.04	0.63	6.0	-	0.06	0.58	-	0.01	0.01	1.93	-	0.009	0.01	5.63	0.16	1.6
**TMS25 (i.p.)**	0.74	0.32	0.01	-	-	-	-	-	0.44	0.28	-	0.13	0.48	0.34	0.005	-	2.07	3.72	0.01	-	100	0.001	0.85
**TMS50 (i.v.)**	0.74	-	0.05	0.008	1.23	-	0.05	0.02	0.86	3.21	0.01	-	0.66	-	0.07	0.1	2.17	-	0.039	0.23	100	0.01	2.3
**TMS50 (i.p.)**	0.47	0.59	0.04	-	-	-	-	-	0.39	0.23	-	0.02	0.26	0.71	0.02	-	2.03	-	0.03	0.18	100	0.01	4.5
**TMS90 (i.v.)**	0.63	-	0.05	0.02	0.92	-	0.05	0.11	0.47	$1.3*10^6$	-	0.09	0.54	-	0.09	0.09	1.72		0.01	0.09	100	0.01	3.8
**TMS150 (i.v.)**	0.67	-	0.15	0.07	1.34	-	0.21	0.15	0.53	-	0.008	0.02	0.50	-	0.12	0.06	2.15	-	0.02	0.5	100	0.01	2.3
**TMS150 (i.p.)**	0.37	0.66	0.06	-	-	-	-	-	0.22	0.91	-	0.04	0.20	0.99	0.05	-	1.63	4.35	0.01	-	100	0.01	1.8
**PEI50 (i.v.)**	0.14	-	0.03	0.03	0.15	-	0.02	0.01	0.17	-	0.003	0.01	0.18	-	0.06	0.03	1.34	-	0.02	0.02	26.3	0.122	3.7
**QA50 (i.v.)**	0.63	-	0.05	0.04	0.99	-	0.04	0.06	0.41	-	0.01	0.03	0.5	-	0.05	0.008	1.87	-	0.04	0.14	75.09	0.053	5.7

Supplementary Table 5 | Group average non-linear regression analysis. Values of model parameter estimates and root mean square error (RMSE) of the fit obtained by fitting the model equations to group average data for various ROIs. Units of A, k_{in}, k_{out}, U_t, and k_u are %ID g^{-1}, h^{-1}, h^{-1}, %ID, and h^{-1}, respectively. (Note: Abdominal aorta and lymph nodes were not analyzed as an ROI in the i.p. and i.v. injection cases, respectively).

Supplementary References

1. Lin, Y.-S. & Haynes, C.L. Impacts of mesoporous silica nanoparticle size, pore ordering, and pore integrity on hemolytic activity. *Journal of the American Chemical Society* **132**, 4834-4842 (2010).
2. Townson, J.L. *et al.* Re-examining the Size/Charge Paradigm: Differing in Vivo Characteristics of Size-and Charge-Matched Mesoporous Silica Nanoparticles. *Journal of the American Chemical Society* **135**, 16030-16033 (2013).
